# Supplementary material for: Substrate-dependent fish have shifted less in distribution under climate change
Source: Commun Biol. 2020 Oct 16;3:586. doi: 10.1038/s42003-020-01325-1 (PMC7567839; doi:10.1038/s42003-020-01325-1)
Supplement: Supplementary file 1 — Supplementary Information [file 42003_2020_1325_MOESM1_ESM.docx]

**Supplemental Figure 1. Historic distribution shifts versus the most important predictor variable for a species distribution in the spring.** Spring shifts in mean centroid (a), percentage change in range size (b), shifts in norther range boundary (c) and southern range boundary (d) from first 5 years and last 5 years in latitudinal degrees versus strongest predictor variable (n=91). Brackets and numbers represent p-value. Whiskers represent 1.5* inter-quartile range. Box represents inter-quartile range as distance between first and third quartiles. Line represents median, red point represents mean, and black points represent outliers (outside of 1.5*IQR).

**b**

**a**

**
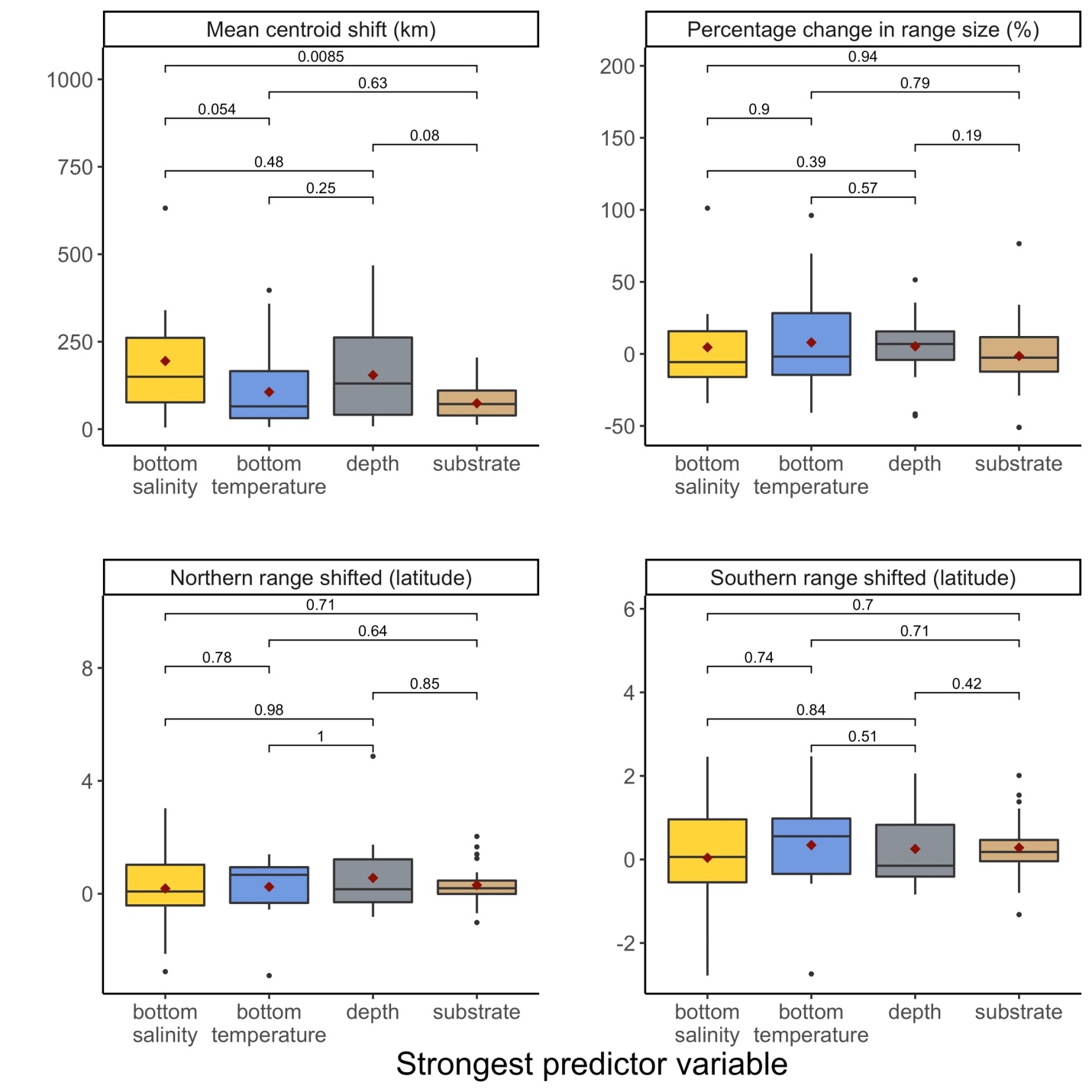
**

**d**

**c**

**The stronger influence of salinity on distribution shifts in the fall is likely due to the combined effect of a drier and warmer weather that has led to more drastic changes in ocean salinity in the fall (less precipitation and warmer weather leads to more evaporation) which most likely effects species distributions more than in the cooler and wetter spring.

**Supplemental Figure 2. Strongest predictor variable for each species group in the spring.** Percentage of each species group that had substrate, depth, bottom temperature, and salinity as the strongest predictor variable in terms of deviance explained for the entire time series (n=91).

**
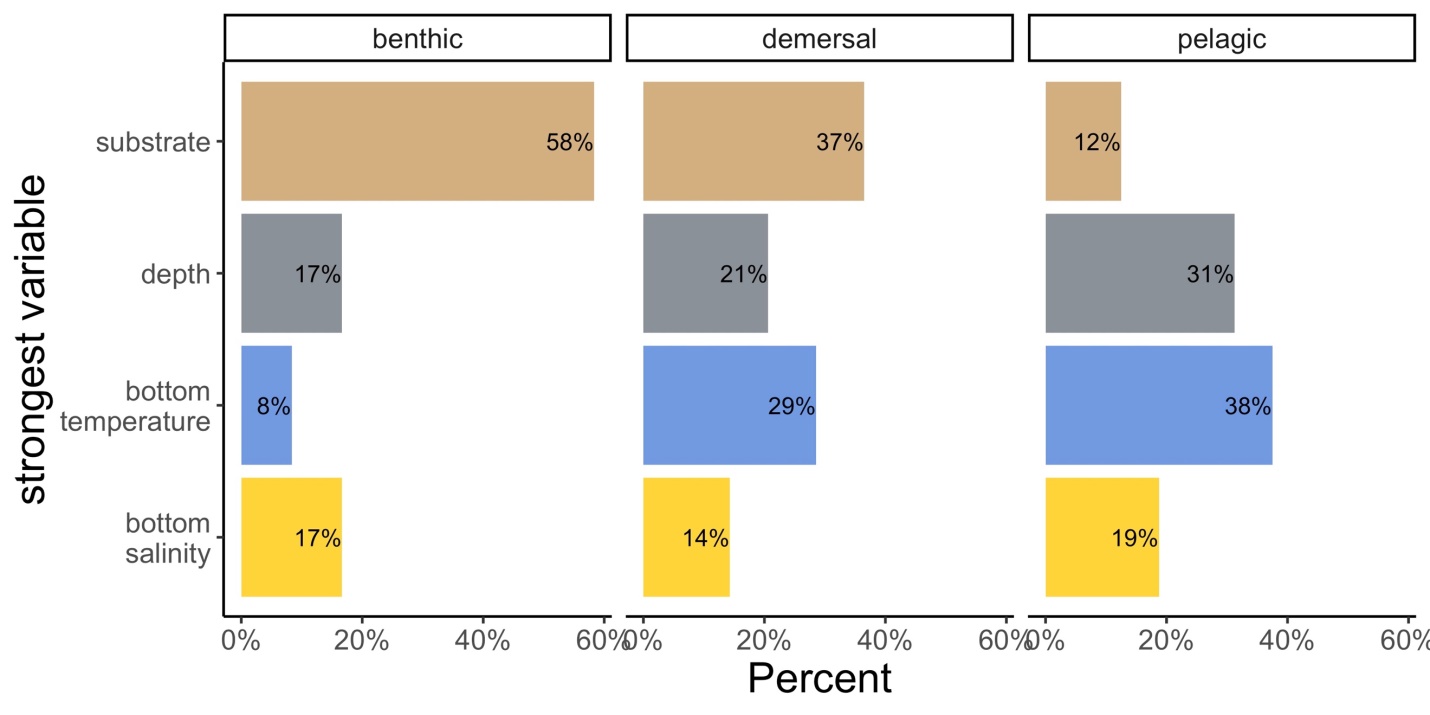
**

**Supplemental Figure 3**. **NEFSC Trawl survey locations between 1986 and 2018**. Trawl locations are shown for the fall (a) and spring (b).

**
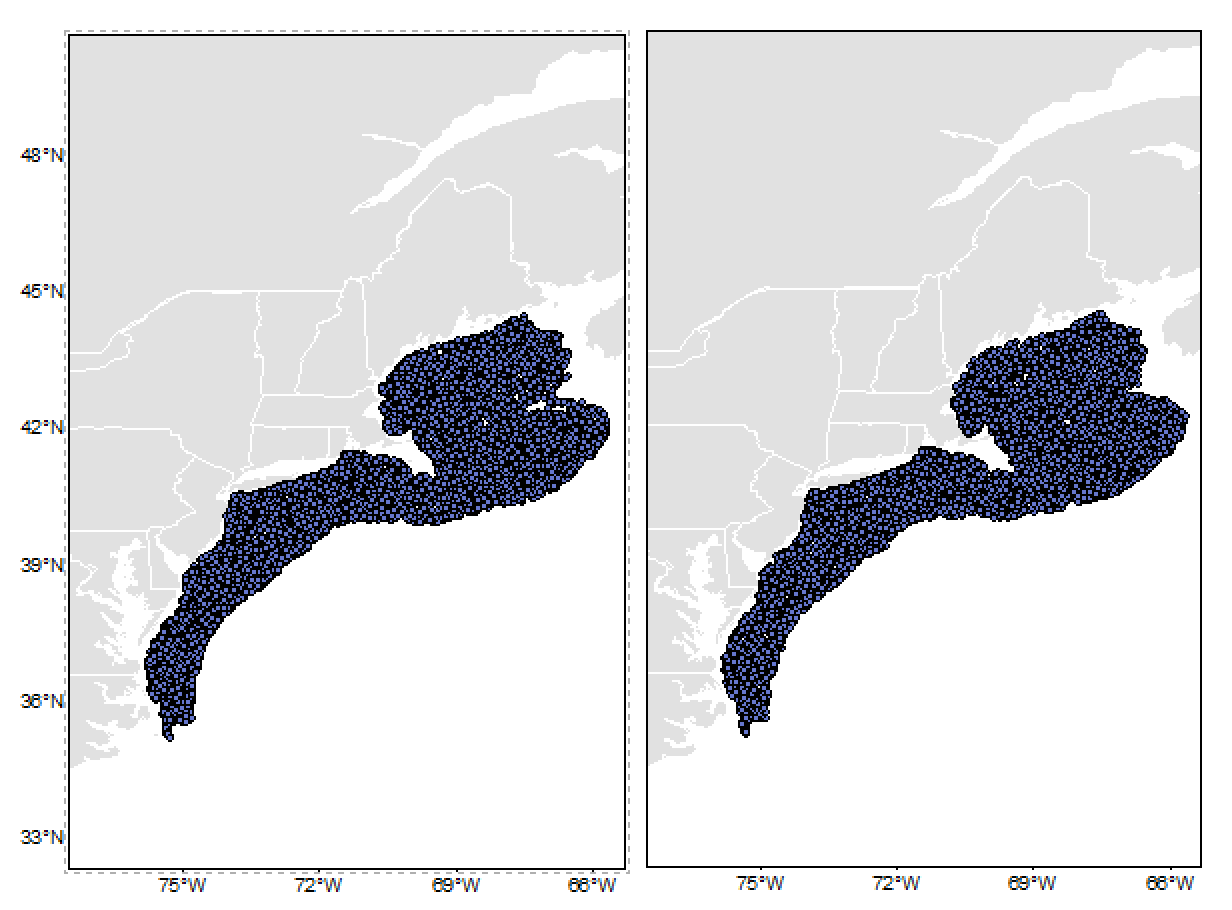
**

a

b

**Supplemental Figure 4.** Substrate categories (sediment grain size) used for GAM analysis, from The Nature Conservancy’s NAMERA study.

**
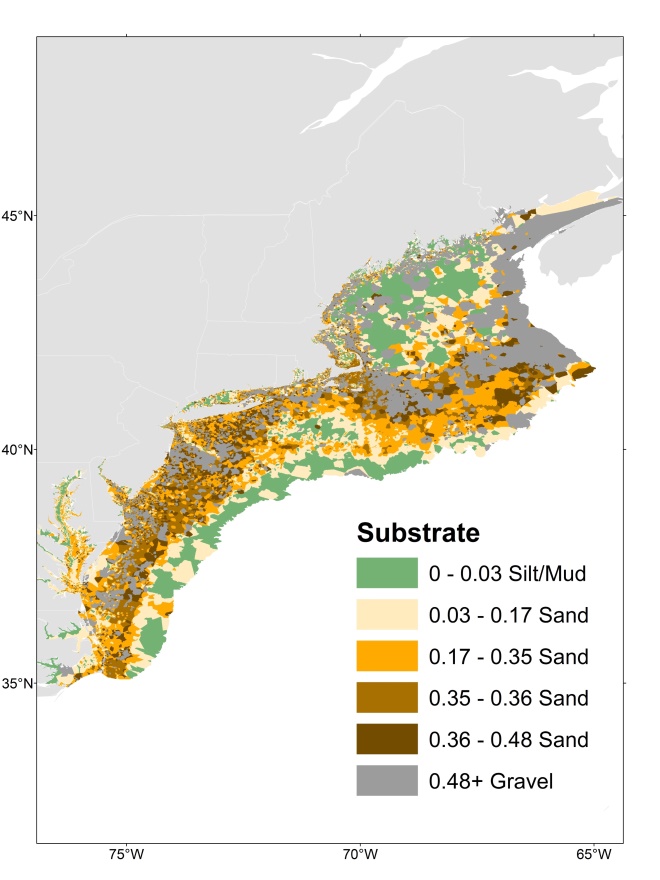
**

**Supplemental Table 1**. Correlation of predictor variables.

| (fall, spring) | Bottom salinity | Bottom temperature | Depth |
| --- | --- | --- | --- |
| Bottom salinity | 1 |  |  |
| Bottom temperature | -0.4, 0.79 | 1 |  |
| Depth | .73, .58 | -0.62, .34 | 1 |
