# Supplementary material for: Substrate-dependent fish have shifted less in distribution under climate change
Source: Commun Biol. 2020 Oct 16;3:586. doi: 10.1038/s42003-020-01325-1 (PMC7567839; doi:10.1038/s42003-020-01325-1)
Supplement: Supplementary file 3 — Description of Additional Supplementary Files [file 42003_2020_1325_MOESM3_ESM.pdf]

Description of Additional Supplementary Files

File Name: Supplementary Data 1
Description: Species selected for study based on the years present and abundance in the first and last five years for the fall. Percentage of deviance explained by each environmental variable from individual model runs vs. null model and strongest predictor variable based on deviance explained for the fall. Species type and geographic shifts of species selected for study in the fall. Total abundance and abundance in the first five and last five years for selected species in the fall.

File Name: Supplementary Data 2
Description: Species selected for study based on the years present and abundance in the first and last five years for the spring. Percentage of deviance explained by each environmental variable from individual model runs vs. null model and strongest predictor variable based on deviance explained for the spring. Species type and geographic shifts of species selected for study in the spring. Total abundance and abundance in the first five and last five years for selected species in the spring
