# Supplementary material for: Substrate-dependent fish have shifted less in distribution under climate change
Source: Commun Biol. 2020 Oct 16;3:586. doi: 10.1038/s42003-020-01325-1 (PMC7567839; doi:10.1038/s42003-020-01325-1)
Supplement: Supplementary file 6 — Reporting Summary [file 42003_2020_1325_MOESM6_ESM.pdf]

nature research

Corresponding author(s): Sarah M. Roberts

Last updated by author(s): 9/2/2020

Reporting Summary

Nature Research wishes to improve the reproducibility of the work that we publish. This form provides structure for consistency and transparency in reporting. For further information on Nature Research policies, see Authors & Referees and the Editorial Policy Checklist .

Statistics

For all statistical analyses, confirm that the following items are present in the figure legend, table legend, main text, or Methods section.

n/a	Confirmed	
☐	☒	The exact sample size (*n*) for each experimental group/condition, given as a discrete number and unit of measurement
☐	☒	A statement on whether measurements were taken from distinct samples or whether the same sample was measured repeatedly
☐	☒	The statistical test(s) used AND whether they are one- or two-sided<br>*Only common tests should be described solely by name; describe more complex techniques in the Methods section.*
☐	☒	A description of all covariates tested
☐	☒	A description of any assumptions or corrections, such as tests of normality and adjustment for multiple comparisons
☐	☒	A full description of the statistical parameters including central tendency (e.g. means) or other basic estimates (e.g. regression coefficient) AND variation (e.g. standard deviation) or associated estimates of uncertainty (e.g. confidence intervals)
☐	☒	For null hypothesis testing, the test statistic (e.g. *F*, *t*, *r*) with confidence intervals, effect sizes, degrees of freedom and *P* value noted<br>*Give P values as exact values whenever suitable.*
☒	☐	For Bayesian analysis, information on the choice of priors and Markov chain Monte Carlo settings
☒	☐	For hierarchical and complex designs, identification of the appropriate level for tests and full reporting of outcomes
☒	☐	Estimates of effect sizes (e.g. Cohen's *d*, Pearson's *r*), indicating how they were calculated

Our web collection on statistics for biologists contains articles on many of the points above.

Software and code

Policy information about availability of computer code

Data collection	Species catch per unit effort (CPUE) data were obtained from the National Oceanographic and Atmospheric Administration (NOAA) Northeast Fishery Science Center (NEFSC) U.S. NES bottom trawl survey, which has been conducted for almost 50 years in the spring and the fall and collected abundance and distribution data for over 250 fish species. The survey employs a stratified random design, with stations allocated proportionally to the stratum area. A 12 mm mesh coded liner is used to retain smaller bodied and juvenile fish, with all fish caught being weighed and counted. We downloaded the data from OceanAdapt which calibrates the CPUE for each species from the different survey ships used. We cleaned the data, excluding certain years as well as species that were not consistently sampled (excluded years prior to 1986 (data begin in 1968) due to irregular sampling of the southern strata, only included strata that were consistently sampled in the spring and fall, and included 93 species in the fall and 91 species in the spring. Species were included if they were present in at least half of the years in both the spring and the fall (16 out of 33 years) and present in the first five years and the last five years in the fall and spring (> 20 CPUE in surveys in 1986-1990 and 2014-2018). We only included fish species (bony fish and cartilaginous fish), as comparing fish species to invertebrates may be inappropriate. We grouped species that inhabit the seafloor as benthic, species that inhabit near the bottom as demersal, and species that inhabit the water column as pelagic (See Supplemental Tables 1 and 2). Groupings were based on McHenry et al's study and Fishbase classifications for additional species26. We compared McHenry et al's classifications to Fishbase and they were similar.<br><br>Ocean temperature, salinity, and depth were collected in situ. Benthic substrate data were obtained from The Nature Conservancy's Northwest Atlantic Marine Ecoregional Assessment (grain size in mm) . Annual, winter, and monthly North Atlantic Oscillation indexes were added based on the year collected, but we removed these variables from the final analysis as the deviance explained was minimal.
Data analysis	All statistical analyses were done using R (R Core Team (2017). R: A language and environment for statistical computing. R Foundation for Statistical Computing, Vienna, Austria. URL https://www.R-project.org/.). We modeled the influence of environmental variables on species CPUE using Generalized Additive Models (GAMs) with a negative binomial error distribution that had a log-link function, penalized regression splines, a REML smoothing parameter with an outer Newton optimizer and 10 knots. We calculated deviance explained by each predictor by running individual GAMs for each variable and species-abundance combination and recorded deviance explained versus the null model. All GAMs were built using the mgcv package in RStudio.

To calculate shifts in species distributions over time we calculated the biomass weighted mean centroid of each species in two time periods: 1986-1990 and 2014-2018 using the spatial Eco package in R26. We calculated shifts in distributions as the geodesic distance between the two biomass weighted mean centroids for each species using the geosphere package in R. We calculated changes in range size using the spatial kernel density function (weighted by population, using gaussian kernels) in the spatial Eco package in R. We calculated each species range as the area with 95% of the populations kernel density for the two time periods as above. Changes in minimum and maximum latitude for each species were calculated using the 95% kernel density range from above, and used the difference between time periods as a measure of change in range size (expansions or contractions in range).

We performed two-sided Wilcoxon nonparametric tests to assess the significance of historical changes in species distributions (mean centroid, range size, minimum and maximum latitude) between the three species groups. We also assessed the significance of deviance explained by each environmental variable between species groups using a two-sided Wilcoxon nonparametric test in RStudio.

For manuscripts utilizing custom algorithms or software that are central to the research but not yet described in published literature, software must be made available to editors/reviewers. We strongly encourage code deposition in a community repository (e.g. GitHub). See the Nature Research guidelines for submitting code & software for further information.

Data

Policy information about availability of data

All manuscripts must include a data availability statement. This statement should provide the following information, where applicable:

- Accession codes, unique identifiers, or web links for publicly available datasets
- A list of figures that have associated raw data
- A description of any restrictions on data availability

All of the data analyzed in this study are publicly available. NEFSC bottom trawl data may be downloaded from OceanAdapt (https://oceanadapt.rutgers.edu). The substrate data can be downloaded from https://www.conservationgateway.org/ConservationByGeography/NorthAmerica/UnitedStates/edc/reportsdata/marine/namera/namera/. Final datasets used to create figures can be downloaded from github (https://github.com/sr197/Sticky_Fish) or Zenodo (DOI http://doi.org/10.5281/zenodo.4000171)

Field-specific reporting

Please select the one below that is the best fit for your research. If you are not sure, read the appropriate sections before making your selection.

☐ Life sciences ☐ Behavioural & social sciences ☒ Ecological, evolutionary & environmental sciences

For a reference copy of the document with all sections, see nature.com/documents/nr-reporting-summary-flat.pdf

Ecological, evolutionary & environmental sciences study design

All studies must disclose on these points even when the disclosure is negative.

Study description	The study used bottom trawl surveys of the North Atlantic, processed by the data originators. Combined with environmental covariates for each study area, the work produced comparisons between pelagic, demersal and benthic species for both their relationship with the environment, and historic distribution and range shifts over the study period. Documentation of the original data collection and subsequent sampling design can be found in https://www.fisheries.noaa.gov/inport/item/22557
Research sample	Species catch per unit effort (CPUE) data were obtained from the National Oceanographic and Atmospheric Administration (NOAA) Northeast Fishery Science Center (NEFSC) U.S. NES bottom trawl survey, which has been conducted for almost 50 years in the spring and the fall and collected abundance and distribution data for over 250 fish species. The survey employs a stratified random design, with stations allocated proportionally to the stratum area. A 12 mm mesh coded liner is used to retain smaller bodied and juvenile fish, with all fish caught being weighed and counted. We downloaded the data from OceanAdapt which calibrates the CPUE for each species from the different survey ships used. We cleaned the data, excluding certain years as well as species that were not consistently sampled (excluded years prior to 1986 (data begin in 1968) due to irregular sampling of the southern strata, only included strata that were consistently sampled in the spring and fall, and included 93 species in the fall and 91 species in the spring (Supplemental Figure 5, Supplemental Tables 1 and 2). Species were included if they were present in at least half of the years in both the spring and the fall (16 out of 33 years) and present in the first five years and the last five years in the fall and spring (> 20 CPUE in surveys in 1986-1990 and 2014-2018). We only included fish species (bony fish and cartilaginous fish), as comparing fish species to invertebrates may be inappropriate. We grouped species that inhabit the seafloor as benthic, species that inhabit near the bottom as demersal, and species that inhabit the water column as pelagic (See Supplemental Tables 1 and 2). Groupings were based on McHenry et al's study and Fishbase classifications for additional species26. We compared McHenry et al's classifications to Fishbase and they were similar.
Sampling strategy	Sampling was based on a stratified random design using area and depth zones. Between 300 and 600 stations were sampled in each survey, with the number of stations allocated to each stratum in proportion to stratum area. Standard tows from 1963-2008 were 30 minutes in duration. Initially, the towing speed was set to approximately 3.5 knots, but in 1996, it was discovered that the speedlog was not working and to go 3.5 knots by the speedlog required a speed of 3.8 knots on the Doppler. Therefore, speed was then changed to 3.8 knots for the rest of the time series. Tow direction was towards the next station unless wind and sea state dictated a different course. For tows in depths >= 183 m, a more specific depth is randomly chosen among four depth intervals and then trawling was done along that depth contour. In 2009, the tows were reduced to 20 minutes in duration and speed was reduced to 3.0 knots. The direction of the tows changed to follow the depth contour.

Data collection	Data were collected by the Northeast FIsheries Science Center. Four vessels conducted the survey, the RV Albatross IV (AL), RV Delaware II (DE), RV Atlantic Twin (AT), and the FRV Henry B. Bigelow (HB), with the AL being the primary survey vessel from 1963-2008. Corsion factors between the AL and DE were calculated for most species. The standard sampling gear used in all autumn surveys was the "#36 Yankee" trawl. The AT surveyed the inshore areas from autumn 1972 to spring 1975 with a ¾ scale "#36 Yankee" trawl rigged with a chain sweep and ground cables, but no conversion factors were calculated for this vessel/gear. In 2009, the AL was replaced with the HB and the #36 Yankee net was replaced with a 4-seam, 3-bridle box net with rock-hopper sweep. Conversion factors for this gear/vessel change were derived for most species.
Timing and spatial scale	Information on the timing and spatial scale of the original data collection can be found here: https://www.nafo.int/Portals/0/PDFs/sc/2014/scr14-024.pdf and here: Azarovitz, T.R. 1994. Northeast Fisheries Science Center Bottom Trawl Surveys. In: Atlantic States Marine Fisheries Commission. 1994. Proceedings of the Workshop on the Collection and Use of Trawl Survey Data for Fisheries Management. Special Report No. 35.<br><br>Summarized below:<br>The NEFSC autumn bottom trawl survey was initiated in 1963. At that time, coverage extended from Nova Scotia, Canada to around Hudson Canyon, NY, at depths between 27 (15 fm) and 366 meters (200 fm). In 1967, strata in the Mid-Atlantic were added (Strata 01610-01760). In 1972, sampling of depths < 27 m (15 fm, strata 03010-03460) was begun for areas south of Massachusetts. In 1979, depths less than 27 m (15 fm) north of Massachusetts were added. Additionally, in 1972, sampling south of Cape Hatteras was conducted all the way south to Florida, restricted to Cape Fear in 1973, not sampled from 1974-1978, sampled to the NC/SC border until 1983 and then reduced to about 14 stations just south of Cape Hatteras. These stations south of Cape Hatteras were not always sampled. In 1987, a stratum in the Bay of Fundy (01350) was split in half into 01351 and 01352, but only the southern portion of the stratum (01351) was ever sampled. When the sampling platform changed in 2009, stations less than 18 m in depth were excluded which eliminated much of the inshore strata and one offshore stratum on the western Scotian Shelf (01330). The survey in 1963 was conducted starting in the northern strata in November and working south to Southern New England in December. When the Mid-Atlantic strata were added, the cruise track changed to start just north of Hudson Canyon, working south offshore to Cape Hatteras, and then working north. This cruise track was fairly stable for the rest of the time series with weather and ship breakdowns affecting the final cruise track. The timing of the start of the survey also changed over time, moving earlier to September. From 2009-2013, the survey timing extended further into November and occasionally into December.<br><br>The spring survey began in 1968 and coverage extended from Nova Scotia to Cape Hatteras at depths > 27 m. In 1973, sampling of depths < 27 m (15 fm, strata 03010-03460) was begun for areas south of Massachusetts. In 1979, depths less than 27 m (15 fm) north of Massachusetts were added. Additionally, in 1974, sampling south of Cape Hatteras in inshore waters was conducted, all the way south to Florida, not sampled again until 1979, but only to the NC/SC border, and in 1986 reduced to about 14 stations just south of Cape Hatteras. In 1987, a stratum in the Bay of Fundy (01350) was split in half into 01351 and 01352, but only the southern portion of the stratum (01351) has ever been sampled. When the sampling platform changed in 2009, stations less than 18 m in depth were excluded which eliminated much of the inshore strata and one offshore stratum on the western Scotian Shelf (01330). The spring survey generally occurs during March and April. There have been some years in which the survey extended until May. In 1973, when the inshore strata were sampled, the AT sampled until May. Ship breakdowns and weather are usually the reasons for late starts or late ends.
Data exclusions	Data were excluded from stratum that were not consistently sampled throughout the time period (sampled every year from 1986-2018).
Reproducibility	All code and data are publicly available to ensure reproducibility.
Randomization	The stratified random sampling design assured for randomization.
Blinding	Not relevant to the statistical study.

Did the study involve field work? ☐ Yes ☒ No

Reporting for specific materials, systems and methods

We require information from authors about some types of materials, experimental systems and methods used in many studies. Here, indicate whether each material, system or method listed is relevant to your study. If you are not sure if a list item applies to your research, read the appropriate section before selecting a response.

Materials & experimental systems

n/a	Involved in the study
☒	☐ Antibodies
☒	☐ Eukaryotic cell lines
☒	☐ Palaeontology
☐	☒ Animals and other organisms
☒	☐ Human research participants
☒	☐ Clinical data

Methods

n/a	Involved in the study
☒	☐ ChIP-seq
☒	☐ Flow cytometry
☒	☐ MRI-based neuroimaging

Animals and other organisms

Policy information about studies involving animals; ARRIVE guidelines recommended for reporting animal research

Laboratory animals	Not applicable
Wild animals	All biological data were collected by the Northeast Fisheries Science Center. Data collection procedures can be found here https://www.fisheries.noaa.gov/inport/item/22557 and here: Azarovitz, T.R. 1994. Northeast Fisheries Science Center Bottom Trawl Surveys. In: Atlantic States Marine Fisheries Commission. 1994. Proceedings of the Workshop on the Collection and Use of Trawl Survey Data for Fisheries Management. Special Report No. 35.
Field-collected samples	All catch were sorted into species and sex, weighed and measured, and catch was disposed at sea after the pertinent data were collected. Original data collection procedures are outlined here: Azarovitz, T.R. 1994. Northeast Fisheries Science Center Bottom Trawl Surveys. In: Atlantic States Marine Fisheries Commission. 1994. Proceedings of the Workshop on the Collection and Use of Trawl Survey Data for Fisheries Management. Special Report No. 35.
Ethics oversight	All biological data were collected by the Northeast Fisheries Science Center. Protected species were handled according to the NOAA and or/NEFSC protocols. Original data collection procedures are outlined here: Azarovitz, T.R. 1994. Northeast Fisheries Science Center Bottom Trawl Surveys. In: Atlantic States Marine Fisheries Commission. 1994. Proceedings of the Workshop on the Collection and Use of Trawl Survey Data for Fisheries Management. Special Report No. 35.

Note that full information on the approval of the study protocol must also be provided in the manuscript.
